# Digital strategies in the global war on myopia

**DOI:** 10.1016/j.lanwpc.2023.100883

**Published:** 2023-08-18

**Authors:** Yih-Chung Tham, Jason C. Yam, Ya Xing Wang, Tien Yin Wong

**Affiliations:** a Centre of Innovation and Precision Eye Health, Department of Ophthalmology, Yong Loo Lin School of Medicine, National University of Singapore and National University Health System, Singapore; b Singapore Eye Research Institute, Singapore National Eye Centre, Singapore; c Eye Academic Clinical Program (Eye ACP), Duke NUS Medical School, Singapore; d Department of Ophthalmology and Visual Sciences, The Chinese University of Hong Kong, Hong Kong, China; e Beijing Ophthalmology and Visual Sciences Key Laboratory, Beijing Institute of Ophthalmology, Beijing Tongren Eye Center, Beijing Tongren Hospital, Capital Medical University, Beijing, China; f Tsinghua Medicine, Tsinghua University, Beijing, China; g School of Clinical Medicine, Beijing Tsinghua Changgung Hospital, Beijing, China

Myopia is a pressing global problem that requires transformative technology to find solutions.^1^, ^2^, ^3^ In recent years, a range of digital health solutions have emerged, offering innovative approaches to tackle both the onset of myopia and halt its progression. However, few studies have thoroughly evaluated the health and economic benefits of these solutions. In this issue of The Lancet Regional Health–Western Pacific, Li et al. present a comprehensive analysis comparing the cost-effectiveness of traditional school-based myopia screening programs versus comprehensive digital prevention and control strategies in China.^4^

The study highlights the potential of digital strategies in myopia management, offering a new perspective on managing myopia in children and adolescents. The authors’ findings suggest that digital approaches can outperform traditional school-based programs in terms of cost-effectiveness, offering a potentially more efficient pathway to combat the escalating public health issue of myopia in China. These insights have far-reaching implications for clinicians, vision scientists, and policymakers and could inform a broader implementation of digital strategies in myopia management.

This work also underscores the need to scale up adoption of digital health platforms, demonstrating that digitized strategies can enhance vision screening coverage and facilitate personalized myopia management. While the key public health strategies of myopia interventions (e.g., increasing outdoor activities, reducing near work, and raising myopia awareness) are known, such interventions require scaling and can be optimally delivered through technology. This ‘shift’ in delivery mode can reach larger audiences, offer immediate intervention, and significantly reduce labor and time costs. To that end, digital communication tools like WeChat and SMS.^5^^,^^6^ are economically viable and sustainable options.

However, it is overly simplistic to suggest that the 'war on myopia' can be won through technological solutions alone. A comprehensive whole-of-society strategy integrating digital, medical, educational, social, and familial interventions is necessary. It is also pertinent to note that the efficacy of digital prevention strategies for myopia, though promising, remains somewhat less compared to other therapies such as low concentration atropines and optical interventions.^5^^,^^7^ Furthermore, addressing other key causal factors, such as educational pressures, excessive near work and screen time, and the impact of urbanization, remains essential.

Li et al.'s research opens another important frontier for future research in myopia prevention and control. Their findings underscore the imperative for nuanced, data-driven insights to ascertain the economic viability of emerging solutions. Nevertheless, while digital strategies hold promise and are cost-effective, their design and implementation require careful consideration. It is crucial that these digital solutions are structured in a bite-sized, user-friendly manner to ensure optimal engagement and efficacy. This is particularly important given the potential paradox in deploying digital interventions for myopia prevention and control. The very risk factor these interventions aim to mitigate–prolonged near work, can be inadvertently exacerbated by increased screen time associated with these digital strategies. If not correctly designed and implemented, these digital strategies could become a double-edged sword. Therefore, in the development and roll-out of digital health interventions, a delicate balance must be struck. This highlights the importance of incorporating implementation science-guided principles into the design and deployment process of these interventions and enhance their real-world effectiveness. Comprehensive digital strategies ought to be guided by user-centric design principles and continuous evaluation, to ensure that the benefits they deliver significantly outweigh any potential drawbacks.

As we pivot towards a future where myopia management is increasingly underpinned by technology, it is critical to ensure equitable access to these digital resources. We must guard against a scenario where disparities in access to digital resources inadvertently exacerbate health inequities. Overcoming barriers related to accessibility, affordability, digital literacy, and cultural appropriateness is paramount. The collective responsibility rests with policymakers, healthcare professionals, technologists, and community and education leaders to actualize this vision of digital health equity in myopia management. In our stride towards improved eye health, it is crucial that no individual or community is left behind.

It is worth noting that the study did not consider certain environmental factors, such as access to green spaces and optimal light exposure. Furthermore, the authors did not evaluate the impact of digital intervention on compliance with other myopia control therapies. Lastly, in light of the increasing prevalence of myopia-related complications, such as pathologic myopia and glaucoma,^3^ evaluating the impact of digital interventions on improving the long-term burden of these complications would be highly informative. Future research should focus on these areas.

In conclusion, findings from this study could be pivotal in the field of myopia prevention and control. If future research further corroborates the greater cost-effectiveness of digital interventions, we could witness a paradigm shift in our approach towards managing this escalating public health issue. These findings reinforce the vital role of data-driven, transformative and innovative strategies in health policy and intervention planning, marking a significant stride towards tackling the myopia epidemic in China and beyond.

## Contributors

Y-CT and TYW conceptualized the paper, wrote the first draft. YXW and JY provided critical revisions and comments to the manuscript. All authors approved the final version of the manuscript.

## Declaration of interests

TYW declares that he is the inventor, patent holder, and co-founder of start-up companies EyRiS and Visre, which have interests in developing digital solutions for eye diseases, including diabetic retinopathy. He also receives consultancy fee from Aldropika Therapeutic, Bayer, Boehringer Ingelheim, Genetech, Iveric Bio, Novartis, Oxurion, Plano, Roche, Sanofi, and Shanghai Henlius.

Other authors declared no competing interest related to this manuscript.
